# The Impact of Social Media Usage on Work Efficiency: The Perspectives of Media Synchronicity and Gratifications

**DOI:** 10.3389/fpsyg.2021.693183

**Published:** 2021-07-28

**Authors:** Din Jong, Shih-Chih Chen, Athapol Ruangkanjanases, Yun-Hsuan Chang

**Affiliations:** ^1^Department of Digital Design and Information Management, Chung Hwa University of Medical Technology, Tainan, Taiwan; ^2^Department of Information Management, National Kaohsiung University of Science and Technology, Kaohsiung, Taiwan; ^3^Chulalongkorn Business School, Chulalongkorn University, Bangkok, Thailand

**Keywords:** social media, media synchronization theory, permanence, publicness, symbol variety, availability, asynchronicity

## Abstract

As prevail of mobile networking, social media became ubiquitous in either work or our personal life. Based on Media Synchronization Theory and transformational framework, this study proposed a research model and examined how the social media' attributes impacting the work effectiveness through the work-oriented or social-oriented usage. The data of 322 valid questionnaires from respondents was analyzed by SmartPLS 3.2.8. The results indicated that the features of social media including availability and symbol variety had the significant influences on their work efficiency through work-oriented usage of social media. Publicness and symbol variety had impact on work efficiency via social-oriented usage of social media. In addition, both social media for work-oriented and social-oriented usage influenced employees' work efficiency. There were different considerations when people selected social media for work or for social purpose. Managers or companies could guide their employees to use the social media in a right way to increase their work features to complete their work efficiency, and create groups for employees so the work information could be shared efficiently.

## Introduction

Social media were electronic tools that enabled users to communicate and exchange information and facilitate interactions among different users (Zerfass et al., 2011; Criado et al., 2013; Song and Lee, 2016). Social media technologies revolutionized the way people communicate and interact socially within and outside of organizations in relation to the Internet, with considerable impact on people's careers and lifestyles (Correa et al., 2010; Turban et al., 2011; Moqbel et al., 2013; Holland et al., 2016). Social media allowed people to communicate or collaborate online through various platforms, weblogs, blogs, wikis, broadcasts, pictures, and videos (Broughton et al., 2009). Social media changed the ways of communication by enabling two-way communication between users rather than one-way.

The social media use at work attracted numerous attentions (van Zoonen et al., 2014a; Van Zoonen et al., 2017). However, most of the researches were in a single perspective (Villanueva et al., 2008), and focused only on social media use (Trainor et al., 2014; Jiang et al., 2016; Parveen et al., 2016; Drummond et al., 2017), or on social media use at work (van Zoonen et al., 2014a; Van Zoonen et al., 2017), on the intensity (Charoensukmongkol, 2014), or on the frequency (Bretschneider and Parker, 2016) of social media use. Some scholars investigated social media use at work mainly on the relationship management (Tajudeen et al., 2018), information search and sharing (de Zubielqui et al., 2019), job satisfaction, and job performance (Parveen et al., 2015).

From the perspective of prior organizational behavior research, social media could be divided into two categories: personal social media and enterprise social media (Van Zoonen et al., 2017). This study emphases on personal social media than enterprise social media for the following reasons: First, there has been extensive research on the use of enterprise social media in the domain of information systems (IS) over the past decade (Leonardi et al., 2013; Leftheriotis and Giannakos, 2014; Huang et al., 2015; Parveen et al., 2015; Bretschneider and Parker, 2016; Hacker et al., 2017; Wehner et al., 2017; Archer-Brown and Kietzmann, 2018; Bulgurcu et al., 2018; Osch and Steinfield, 2018; de Zubielqui et al., 2019; Fu et al., 2019; Veeravalli and Vijayalakshmi, 2019; Tamengkel and Rumawas, 2020). Some studies discussed the impact of enterprise social media use in organizations, such as organizational rules, norms, and policies, organization type, and size (Bretschneider and Parker, 2016). The other studies investigated whether the use of enterprise social media in organizations could facilitate internal knowledge management (Behringer et al., 2017; Kane, 2017; Bulgurcu et al., 2018), communication efficiency (Korzynski, 2014), cross-nation social networking (Van Osch and Steinfield, 2016), strategic vision of communicators (Charoensukmongkol, 2014), perceived values of utilitarianism and hedonism (Leftheriotis and Giannakos, 2014), innovation (Lam et al., 2016; Kapoor et al., 2018; Papa et al., 2018), job satisfaction (Charoensukmongkol and Sasatanun, 2017; Song et al., 2019), relationship satisfaction (Sheer and Rice, 2017), job performance improvement (Charoensukmongkol and Sasatanun, 2017; Song et al., 2019), organizational performance (Garcia-Morales et al., 2018), or corporate performance (de Zubielqui et al., 2019; Nisar et al., 2019). Second, unlike enterprise social media, which is strictly limited used by organizational employees, personal social media was available for everyone. That meant that personal social media could easily bridge the gap between personal and professional lives. The use of personal social media not only allowed employees to communicate and connect with their families or handle family matters at work, but also let employees to receive and complete work assignments after working hour, in the evening or on the weekends when at home (Moqbel et al., 2013). Therefore, in synthesis with above discussion, this study would emphasize to evaluate and explain the impact of different characteristics of social media on work efficiency through the work-oriented and social-oriented usage intention of social media.

## Literature Review

### Uses and Gratifications Theory

Uses and Gratifications Theory (UGT) was a mass communication theory (Eighmey and McCord, 1998) that had been applied to traditional media to understand customer behavior. Uses and Gratifications Theory explained the origin of social and psychological needs that generated expectations of the media, thus created different patterns of media exposure or involvement in other activities that lead to satisfaction of needs (Katz et al., 1973). Uses and Gratifications Theory has received considerable attention in social media research, especially in the satisfaction of customer' needs (Dholakia et al., 2004; Porter and Donthu, 2008; Chen, 2010).

In recent years, scholars used the UST to explain individuals' social media use and demand satisfaction. For example, Ali-Hassan et al. (2015) conceptualized demand and satisfaction theory through three dimensions of social media use, including demand, job innovation, social use, hedonic use, and cognitive use, and examined their effects on practitioner performance. Their findings indicated that the use of social and cognitive technologies positively affected employees' daily work and innovative work, while the use of hedonic technologies negatively affected daily work. Based on the UGT, Odoom et al. (2017) found that the use of social media positively influenced the performance gains that companies received, and UGT helped to explain why people choose and respond to different types of media and information when faced with numerous media and messaging options (Xu et al., 2019). The principle of UGT to explain user behavior was that media use was selective and self-conscious, motivated by individuals' rational needs. The expectation of their needs would be met through specific types of media or content (Ruggiero, 2000). Since the UGT provided a link between choice and outcome, therefore, it was appropriate for the study to explore the effects of social media use on productivity.

### Social Media Use

Social media could be used for either social or work-related purposes in enterprises (Gonzalez et al., 2013). Social media such as WeChat was widely used for work-related purposes in Chinese enterprises (Zhang et al., 2018). In Taiwan, Apps such as Line or Facebook Messenger are common to be used in the workplace. Based on the UGT, Liang et al. (2020) conceptualized the employee' needs of using social media into two dimensions: work-oriented and social-oriented. Their study confirmed that employees would use social media for social-related or work-related purposes. The use of social-related motives promoted employee job satisfaction, while the use of work-related motives increased employee productivity.

Specifically, social-oriented usage of social media was defined as the use of social media to establish new social relationships like making new friends, to identify individuals with common interests, and to maintain contact with existing friends and customers. Work-oriented usage of social media was defined as using social media to discuss work with colleagues, or to share document and file information within the organization. Since the UGT provides a link between usage choices and their outcomes (Liang et al., 2020), UGT could be considered as a framework for understanding the relationship between motivation and productivity in the media use (Stafford et al., 2004; Ali-Hassan et al., 2015).

According to the UGT, employees achieved satisfaction when they chose a specific media that could meet their needs. Social media had significant impacts on various communication or management in either workplaces or businesses. Previous studies had shown that the use of social media in organizations could facilitate internal knowledge management (Korzynski, 2014; Behringer et al., 2017; Charoensukmongkol and Sasatanun, 2017; Kane, 2017), and increased communication efficiency, and even enhance work performance. Therefore, this study extended the work of Liang et al. (2020) to classify the type of social media use for employees, and explored how the characteristics of social media affected the work efficiency. This would bridge the gap between theory and practice and provide reference for corporate decision making.

### Media Synchronicity Theory

Media Synchronicity Theory (MST) by Dennis et al. (2008) suggested that synchronization existed between people when they worked together. Media Synchronicity Theory identifies five objective capabilities that could affect the level of synchronization:

- Transmission speed: the speed at which the media can transmit messages.
- Parallel processing: the degree to which the media can transmit messages from multiple senders simultaneously.
- Symbol diversity: the number of ways in which information can be conveyed.
- Rehearsal: the degree to which the communication media allows senders to rehearse or adjust messages before sending; and
- Re-processing: the degree to which messages can be rechecked or reprocessed by the recipient.

In addition, Dennis et al. (2008) proposed that all tasks were composed of two communication processes: conveyance and convergence. The conveyance process focuses on the exchange of large amounts of new information, while the convergence process involves consensus on the information already processed. Media Synchronicity Theory attempts to determine the ideal match between media capabilities and communication processes in terms of achieving optimal communication performance. In addition to explaining how different media capabilities affected the effectiveness of communication, Media Synchronicity Theory also examined the differences in the communication process and the degree to which individuals must be involved in the transmission and processing of messages in order for communication to be successful.

## Research Methods

### Research Hypotheses

The literature review on enterprise-based social media use indicates that social media use can enhance work performance (Wu, 2016; Brooks and Califf, 2017; Moqbel and Nah, 2017; Tamengkel and Rumawas, 2020), organizational performance (Parveen et al., 2015; Tajvidi and Karami, 2017; Garcia-Morales et al., 2018; Nisar et al., 2019), situational performance (Trainor et al., 2014; Ng et al., 2016), routine and innovative performance (Ali-Hassan et al., 2015; Kuegler et al., 2015; Ng et al., 2016). For example, prior studies examined the potential social, hedonic, and cognitive outcomes when employees used personal-based social media (Ali-Hassan et al., 2015; Ali et al., 2019; Cao and Yu, 2019). Liang et al. (2020) showed that employees would use personal or corporate social media for work and social-related purposes. The use of social-related motives can promote employee job satisfaction, and work-related motives can increase employee productivity. Therefore, the following hypothesis is proposed:

H1: Work-oriented usage of social media positively affects work efficiency.

Work efficiency is the ratio of labor output to time invested in an event (Sickles and Zelenyuk, 2019). Previous researches focused on productivity increasement (Liang et al., 2020; Priyadarshini et al., 2020; Vithayathil et al., 2020), and the factors that influenced productivity (Sutanto et al., 2018). Regarding the relationship between social media use and work productivity, studies has shown that work-related social media use could enhance the quality of communication and information exchange among employees, which in turn positively affected their work productivity (Leftheriotis and Giannakos, 2014).

Social media for social-oriented usage is to exchange personal information in a social manner, and to gain social and emotional support through the expression and connection of one's identity. When employees used social media for social-related purposes, they generated online communication and social interaction. Employees' motivation for using social media was primarily to observe the market (i.e., data collection), and secondarily to maintain contact with customers (i.e., strengthening contacts) (Leftheriotis and Giannakos, 2014). Based on the above discussion, the following hypotheses were proposed:

H2: Social-oriented usage of social media positively affects work efficiency.

Media synchronization theory was used to describe and evaluate physical media functions (Muhren et al., 2009; Davison et al., 2014). This theory identified five physical media functions that may affect media synchronization. They were 1. transmission speed, 2. parallel processing, 3. symbol diversity, 4. rehearsability, and 5. reprocessing. Previous studies found that the functions of social media had impact on work performance (Leftheriotis and Giannakos, 2014; Wang et al., 2016; Salehan et al., 2017). Based on the social media features proposed by Nesi et al. (2018), this study consolidated them into five social media features that may affect the motivation of social media use: asynchronicity, work efficiency, publicness, accessibility, and symbol variety.

The aspect of asynchronicity has long been emphasized in the study of psychology or media influence (Valkenburg and Peter, 2011; McFarl and Ployhart, 2015). Berger (2013) stressed the inherent asynchronous nature of non-verbal communication, which is more prevalent in social media. Social media varied in the response time when communication. For example, video communication provided nearly perfect synchronization, whereas email was in an asynchronous manner, leaving more time for the user to read or construct the message to be replied to. Although some researches treated instant messaging as a synchronous communication, Münzer and Borg (2008) suggested that social media often could not provide immediate interpersonal feedback (e.g., the time interval in constructing the message).

As described in media synchronization theory (Dennis et al., 2008), the media for communication should have a variety of functions, including the speed at which messages are delivered (transmission speed), the degree to which interactions can occur simultaneously (parallel processing), and the degree to which messages can be crafted (rehearsability). As one of the basic functions of social media was for social-oriented usage, it could fulfill the need for employees to create and maintain social relationships through social networking or communities of interest (Wu, 2013). Social media can connect individuals with family, friends, associates, or colleagues anytime, anywhere. As the number of social relationships embedded in social networks grows, employees might receive a large number of messages from their virtual friends in social media. In order to maintain a large social network for gaining support and belonging, individuals might frequently check their social media to respond messages as quickly as possible (Cao et al., 2016). In light of the above studies, the following hypotheses were proposed:

H3a: Asynchronicity negatively affects social media for work-oriented usage.
H3b: Asynchronicity negatively affects social media for social-oriented usage.

Permanence referred to the extent to which content or messages remained accessible after interaction or posted (McFarl and Ployhart, 2015). Media with permanence feature could automatic record or archive things presented online. User must be aware of the permanence feature of social media before posting content, because social media like Facebook that posted photos could be searched years later. However, social media like Instagram, the posted content would be removed from other users' cellphones in 24 h after it was sent. No matter these posted contents could be retrieved or erased, viewers could easily snapshot the screen and stored it. This study proposed that permanence is a driving force for social media use, because of its searchability (Boyd, 2010), retrievability and replicability (Boyd, 2010; Peter and Valkenburg, 2013). Similarly, permanence gave the users the opportunity to re-examine previously shared content—reprocessing (Dennis et al., 2008), and to examine or verify information—verifiability (McFarland and Ployhart, 2015). Thus, permanence is a broadly encompassing feature of social media that is described in previous discussions (Dennis et al., 2008; Peter and Valkenburg, 2013; McFarland and Ployhart, 2015). The following hypotheses are presented.

H4a: Permanence positively affects social media for work-oriented usage.
H4b: Permanence positively affects social media for social-oriented usage.

Social media allowed information to be shared within a large group of people simultaneously. McFarl and Ployhart (2015) described this phenomenon as interdependent. Since the content was not send to designated recipients, some studies focused on larger audiences or potentially invisible audiences (Berger, 2013). The function of the social media was referred as publicity because workers could communicate publicly with their supervisory colleagues, customers, or even strangers that could not be done offline. For employees to promote or publicize their personal information might met the expectation of their audiences (Boyd, 2014; Underwood and Ehrenreich, 2017).

It is obvious for some social media activities that has the public nature (e.g., posting photos on Instagram or Snapchat). The public nature can also occur in forums or LINE groups, etc. For example, in thread forums or group chats, people can easily communicate with 10–20 friends or more groups at the same time. For employees, promoting or publicizing their personal information might create audiences and satisfied their expectation (Boyd, 2014; Underwood and Ehrenreich, 2017).

The majority of studies had explicitly declared that computer-mediated communication as a relatively more private way to obtain or provide support for team communication (Wright, 2015). Comparing with online support groups, communication in the community had a higher degree of publicness, in means of that the possibility that one person's behavior will be observed by others or may learn the number of other perpetrators (Leary and Kowalski, 1990).

Public announcements on social media can attract a wider audience, expand the space for interpersonal communication, and redefine the context in which support is sought and given (Treem and Leonardi, 2013). Given the different influences of users on interpersonal relationships, this may further affect the outcome of users seeking support on social media (Bazarova, 2012; Liu and Kang, 2017). In the social media communication environment, publicness could change the way users viewed their empathy or support from their audiences, or affect the likelihood of providing support on social media externally (Liu and Wei, 2018). Under the working environment setting, employees might want to disclose their personal information, moods, etc., on the social media to connect more people or customers. Therefore, the following hypotheses are proposed:

H5a: Publicness positively affects social media for work-oriented usage.
H5b: Publicness positively affects social media for social-oriented usage.

The availability was defined as the ease of posting or sharing content regardless of its physical location. The accessibility provided the possibility of easily initiating connections or joining social networks, which greatly facilitates the ease of social media communication (Valkenburg and Peter, 2011; McFarl and Ployhart, 2015). For example, picking up the phone or sending a text message to friends requires less effort than driving to a friend's house and talk. Similarly, it needs much less effort chatting with strangers online than attending a party to meet someone new. Employees in certain industries requires extensive and strong social networks. The higher the demand for human interaction, the more frequent the relationships and connections need to be.

The media synchronization theory had emphasized that social media synchronization affects social intimacy (Park et al., 2019). Given the focus on the impact of social media on worker efficiency, this study believed that employees' ability to quickly access or share content with customers was a result of availability. In conjunction with publicness, the availability of specific social media could enable “scalability.” That has the potential for content to be highly visible, through reposting a “fast-moving” message or video (Boyd, 2010). Therefore, the following hypotheses were proposed:

H6a: Availability positively affects social media for work-oriented usage.
H6b: Availability positively affects social media for social-oriented usage.

Symbol variety represented the various ways the media have to encode information for communication (Dennis et al., 2008). People use different types of symbols to convey meanings in the communication process. Therefore, symbol variety is of paramount importance. In face-to-face conversations, people could communicate in a variety of ways, such as handshakes, facial expressions, head movements, and tone of voice. However, text-based real-time communication such as SMS services were relatively limited, as cue absence was one of the characteristics of social media (Nesi et al., 2018). Cue absence originated from the theory of cue filtering in computer-mediated communication (Culnan and Markus, 1987) and the concept of anonymity and social presence described in various fields (Subrahmanyam and Šmahel, 2011; Valkenburg and Peter, 2011; Berger and Iyengar, 2013; McFarland and Ployhart, 2015). In social media, the aspects that lack of physical presence such as voice, body touch, gestures, and facial expressions, excluded the possibility of interpersonal cues/clues, and reduced the amount of message or symbol variety.

Media synchronization theory found that the media with higher symbol variety provided higher perceptual interaction during communication because it took the least time and effort to encode and decode messages (Dennis et al., 2008). The symbol variety of social media contains multiple symbols of text, video and audio with a variety of features that provide users with enhanced functionality. It complements the missing cues, thus minimizing confusion and uncertainty in communication. Therefore, people could avoid unexpected misunderstandings and create a harmonious communication environment, thus enhancing inter-personal intimacy (Tang et al., 2013). Thus, the following hypotheses were proposed:

H5a: Symbol variety positively affects social media for work-oriented usage.
H5b: Symbol variety positively affects social media for social-oriented usage.

**Figure 1:**
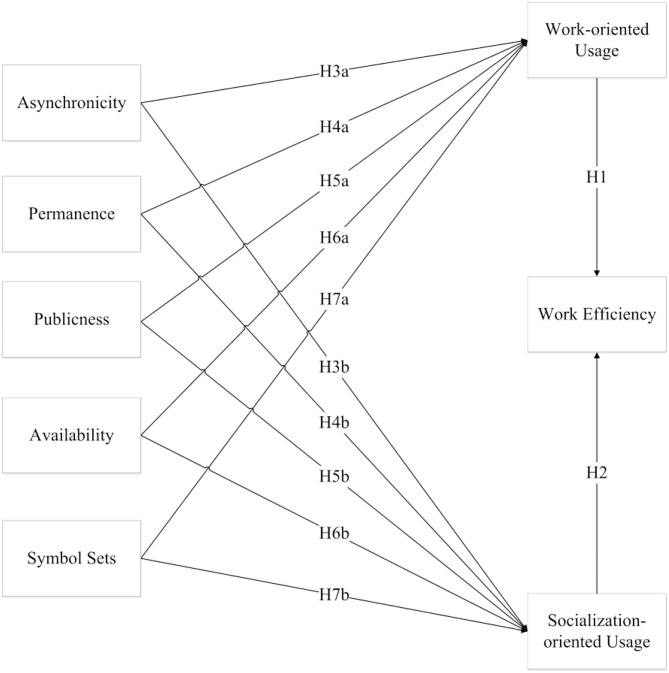
Research model and hypothesis.

The purpose of this study is to investigate the effects of social media features on work efficiency. Based on previous studies, the social media use either for work or for social was summarized. In order to understand the relationship between several configurations, several hypotheses were proposed and examined in Figure 1.

### Research Subjects and Data Collection

The respondents were those who had experience in using social media such as Facebook, Instagram, Facebook Messenger, Line, Whatsapp, or Wechat in Taiwan. A screening question was set at the beginning of the questionnaire (as shown in Appendix Table A1) to ensure that only respondents with experience that using social media at work could participate in the survey. The survey was conducted in the end of 2020, and data were collected anonymously. After removing 7 invalid responses, a total of 322 questionnaires were collected. Partial least square structural equation model (PLS-SEM) was widely used in various research fields and could be used to perform simultaneous cross-construct measurements and structural model tests (Chin et al., 2003). Partial least square structural equation model was suitable for relatively early theoretical development studies, and it was possible to process statistical analyses between study sections and variables with more robust parametric results than other statistical methods, even with small or medium-sized samples (Chin, 1998; Chin et al., 2003). The summarized information of the respondents was shown as Table 1.

**Table 1:** Sample demographic.

**Attribute**	**Types**	**Sample (*N* = 322)**	**Percentage (%)**
Sex	Male	174	54
	Female	148	46
Age	20 and under	18	6
	21–30	176	54
	31–40	64	20
	41–50	45	14
	51 and above	21	6
Social media used in work	Line app	287	40
	Facebook	158	22
	Instagram	116	16
	Facebook Messenger	98	14
	Wechat	44	6
	Whatsapp	19	3

## Results

This study used PLS to conduct a validated factor analysis (CFA) to extract the average variables extracted (AVE) for the construct questions, compose reliability values (CR) and Cronbach's alpha (Gefen et al., 2000) to assess the convergent validity and to measure the reliability of this reliability of the study questions.

### Model Reliability and Validity Analysis

The results of the factor loadings and reliability tests for each of the study's constructs were summarized in Table 2. The AVE values were greater than the recommended value of 0.5 (Fornell and Larcker, 1981; Gefen et al., 2000), and the Cronbach's alpha values and composite reliabilities for all constructs were >0.7, meeting the criteria for academic studies (Fornell and Larcker, 1981; Nunnally and Bernstein, 1994; Gefen et al., 2000). Therefore, the convergent validity and reliability of the measurement model passed the examination.

**Table 2:** Reliability tests for constructs and items.

**Constructs**	**Items**	**Factor loadings**	**Cronbach's alpha**	**Composite validity (CR)**	**Average variance extracted (AVE)**
Asynchronicity	ASY1	0.788	0.723	0.843	0.644
	ASY2	0.757			
	ASY3	0.859			
Permanence	PER1	0.893	0.848	0.908	0.768
	PER2	0.891			
	PER3	0.843			
Publicness	PUB1	0.881	0.885	0.929	0.813
	PUB2	0.925			
	PUB3	0.897			
Availability	AVA1	0.892	0.902	0.939	0.836
	AVA2	0.925			
	AVA3	0.926			
Symbol variety	SYM1	0.850	0.824	0.885	0.660
	SYM2	0.668			
	SYM3	0.875			
	SYM4	0.841			
Social-oriented usage of social media	SOC1	0.877	0.870	0.913	0.724
	SOC2	0.876			
	SOC3	0.907			
	SOC4	0.734			
Work-oriented usage of social media	WOR1	0.833	0.868	0.910	0.717
	WOR2	0.897			
	WOR3	0.864			
	WOR4	0.789			
Work efficiency	WEF1	0.954	0.912	0.945	0.851
	WEF2	0.950			

In this study, both convergent validity and discriminant validity tests were conducted. According to Fornell and Larcker (1981), the factor loadings of variables >0.5, the average variable extraction (AVE) must be >0.5, and the reliability must be >0.7. From Table 3, it indicated that all constructs in this study had convergent validity. The square root of AVE for each construct was greater than the correlation coefficient between the constructs, therefore all constructs in the measurement model had discriminant validity (Fornell and Larcker, 1981).

**Table 3:** Correlation coefficient matrix between latent variables.

**Constructs**	**Asynchronicity**	**Permanence**	**Publicness**	**Availability**	**Symbol variety**	**Social usage**	**Work usage**	**Work efficiency**
Asynchronicity	**0.802**							
Permanence	0.623	**0.876**						
Publicness	0.397	0.225	**0.901**					
Availability	0.681	0.581	0.402	**0.915**				
Symbol variety	0.392	0.388	0.282	0.461	**0.813**			
Social usage	0.387	0.358	0.332	0.411	0.39	**0.851**		
Work usage	0.516	0.458	0.362	0.74	0.468	0.477	**0.847**	
Work efficiency	0.593	0.484	0.358	0.744	0.45	0.434	0.723	**0.923**

*The bold diagonal value is the square root of the AVE of each latent variable*.

### Hypothesis Tests and Path Analysis

**Figure 2:**
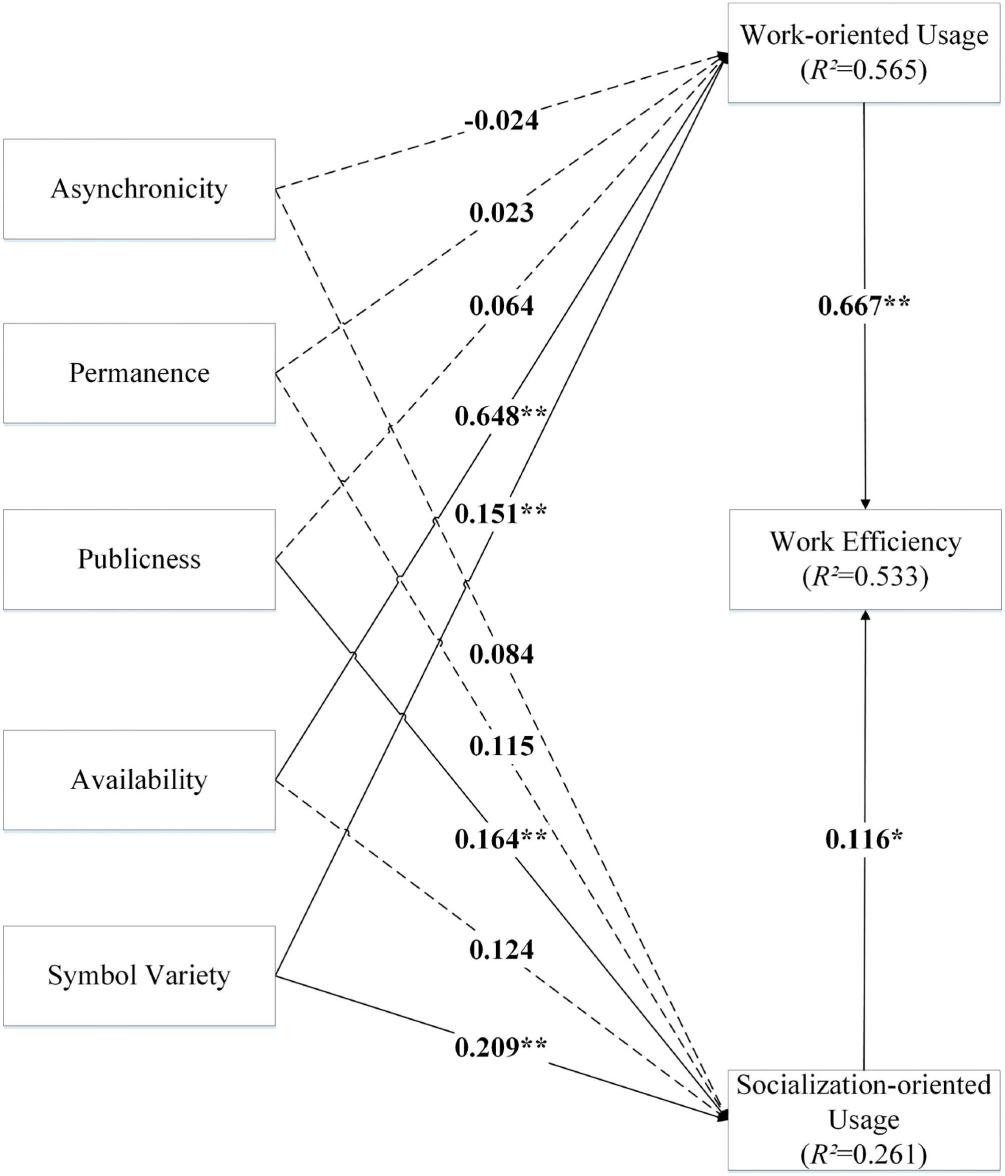
Result of path analysis. Note: *p-value < 0.05; **p-value < 0.01.

In this study, SmartPLS 3.2.8 performs structural pattern analysis. The results of the path analyses were shown in Figure 2, and the hypothesis test results were in Figure 1. At 95% confidence level, 6 of the 12 proposed research hypotheses were supported.

The results showed that all hypotheses were supported except hypotheses 3a, 3b, 4a, 4b, 5a, and 6b, which were not supported (as shown in Table 4). Specifically, the impact of work-oriented usage (*t* = 12.933, *p* < 0.01) and social-oriented usage (*t* = 2.287, *p* < 0.05) on work efficiency were positively correlated. Regarding the effect of social media features on work use, only symbol variety (*t* = 4.195, *p* < 0.01) was positively related to work use, while asynchronicity (*t* = 0.390, *p* > 0.10), permanence (*t* = 0.385, *p* > 0.10), publicness (*t* = 1.418, *p* > 0.10), and availability (*t* = 1.455, >0.10) had no significant effect on work use. About the effect of social media features on social-oriented usage of social media, only publicness (*t* = 2.921, *p* < 0.01) and symbol diversity (*t* = 3.064, *p* < 0.01) were positively related to social-oriented usage, while asynchronicity (*t* = 1.042, *p* > 0.10), permanence (*t* = 1.683, *p* > 0.10) and availability (*t* = 1.455, *p* > 0.10) had no significant influence on social-oriented usage of social media.

**Table 4:** Hypotheses tests.

**Hypotheses/Structural path**	**Path coefficient**	***t*-Value**	***P*-value**	**95% Confidence interval**	**Results**
H1: Work usage → work efficiency	0.667^**^	12.933	0.000	(0.558, 0.759)	Supported
H2: Social usage → work efficiency	0.116^*^	2.287	0.022	(0.019, 0.217)	Supported
H3a: Asynchronicity → work usage	−0.024	0.39	0.697	(−0.145, 0.094)	Not supported
H3b: Asynchronicity → social usage	0.084	1.042	0.297	(−0.068, 0.251)	Not supported
H4a: Permanence → work usage	0.023	0.385	0.700	(−0.089, 0.146)	Not supported
H4b: Permanence → social usage	0.115	1.683	0.092	(−0.026, 0.242)	Not supported
H5a: Publicness → work usage	0.064	1.418	0.157	(−0.018, 0.151)	Not supported
H5b: Publicness → social usage	0.164^**^	2.921	0.004	(0.058, 0.270)	Supported
H6a: Availability → work usage	0.648^**^	10.965	0.000	(0.527, 0.753)	Supported
H6b: Availability → social usage	0.124	1.455	0.146	(−0.036, 0.283)	Not supported
H7a: Symbol variety → work usage	0.151^**^	4.195	0.004	(0.056, 0.259)	Supported
H7b: Symbol variety → social usage	0.209^**^	3.064	0.002	(0.076, 0.350)	Supported

*Note:*

* *p-value < 0.05;*

** *p-value < 0.01*.

### Research Findings and Discussion

The results supported hypothesis 1 that the social media for work use has a significant impact on work efficiency. This finding suggests that practitioners' work efficiency can be improved when using social media as a workplace tool. This conclusion is consistent with previous research on the use of social media in the workplace (Wu et al., 2006; Mansi and Levy, 2013).

The results of this study indicated that hypothesis 2 is supported. Socially oriented social media use, such as casual conversations with colleagues, can lead to smoother social interactions and increased awareness of social capital (Ali-Hassan et al., 2015), leading to an increase in utilitarian use (Song et al., 2019). Practitioners can use social media to meet new people or even to explore new clients to increase work proficiency.

Hypothesis 5b that publicness has a positive impact on social-oriented usage of social media was supported. Social media users can take advantage of the publicness to present themselves. They can also browse other users' public information to find communities or groups with similar interests, and make new friends or meet other people who are not easy to meet in real life. Thus, publicness has a positive effect on social media for social usage.

Hypothesis 6a that availability has a positive effect on social media for work use. However, hypothesis 6b that availability has no positive influence on the social media for social usage. Availability in social media allows practitioners to connect and join other communities easily. However, it is possible that this social media characteristic of “being able to easily connect with customers” causes some practitioners to view it as part of their job. Therefore, availability has a positive effect on practitioners for work purposes, but not significantly enough for social use.

Hypothesis 7a and 7b were both supported that symbol variety has significant impact on social media for both work-oriented and social-oriented usage. Different social media provides diverse services. The social media with limited symbol variety can send text-only messages or photos that provide less interpersonal cues (no facial expressions, tone of voice, or gestures). Previous study finds that the level of perceived symbol variety in non-enterprise social media positively influences users' use for both social and work purposes. When people use instant messaging for either personal or business purposes rather than for specific purposes, the use of emojis and photo images can increase social intimacy between the communicating parties (Park and Lee, 2019).

## Limitations

Several research limitations are shown as follows. First, all participants in this study were from Taiwan, and it is uncertain whether our findings can be generalized to other countries. Moreover, the online survey instrument in this study was intended to distribute to the employees who use social media. However, the answers from the respondents might not reflect the situation set by the purpose of the study. Second, the inference of the results may be limited because of the features of different social media. In this study, non-enterprise social media, such as Facebook, Instagram, Line App, etc. were the main social media investigated. However, a more private concerned corporate social media, such as Skype, Slack, etc., which may bring different results due to their different features. Third, although some studies attempted to identify the antecedents and consequences of social media use in enterprise (Parveen et al., 2015; Jiang et al., 2016), most of these studies treat employees as homogeneous entities and ignore the potential group differences (Krasnova et al., 2017). Earlier research has found significant gender differences in IT social media use patterns (Muscanell and Guadagno, 2012). This suggested that the outcomes of social media use in the enterprise may also differ between male and female employees. Finally, this study categorized social usage and work usage as the application of social media by practitioners. In fact, the motivation of social media use could be divided into different categories, such as hedonic needs and knowledge needs (Ali-Hassan et al., 2015). Future research could explore the multiple effects of social media use for the other purposes and examine the results. Finally, this paper examined the direct relationship between social media use and work efficiency, but did not explore the process between independent variables and outcome variables. Other mediating variables related to the use of social media might influence the results of the study.

## Data Availability Statement

The raw data supporting the conclusions of this article will be made available by the authors, without undue reservation.

## Ethics Statement

Ethical review and approval was not required for the study on human participants in accordance with the local legislation and institutional requirements. Written informed consent for participation was not required for this study in accordance with the national legislation and the institutional requirements.

## Author Contributions

DJ: conceptualization, methodology, data curation, and writing—review and editing. S-CC: formal analysis, and supervision. Y-HC: investigation. AR: writing—original draft preparation. DJ, S-CC, AR, and Y-HC: validation. All authors contributed to the article and approved the submitted version.

## Conflict of Interest

The authors declare that the research was conducted in the absence of any commercial or financial relationships that could be construed as a potential conflict of interest.

## Publisher's Note

All claims expressed in this article are solely those of the authors and do not necessarily represent those of their affiliated organizations, or those of the publisher, the editors and the reviewers. Any product that may be evaluated in this article, or claim that may be made by its manufacturer, is not guaranteed or endorsed by the publisher.

## Figures and Tables

**Table 5:** Measurement items.

**Construct**	**Item**	**Measurement**
Asynchronicity	ASY1	I can receive replies from my clients immediately after sending them a message via social media.
	ASY2	I reply as soon as I receive a message in social media.
	ASY3	Social media can help me communicate with customers immediately.
Permanence	PER1	I can read past messages to clearly understand the previous conversations with customers.
	PER2	I read past messages to help recall previous conversations with clients.
	PER3	If the message is very long and complicated, I can read the message carefully.
Publicness	PUB1	I don't think the personal information disclosed on social media affects my current work.
	PUB2	I don't think the personal information disclosed on social media affects the perception of my customers.
	PUB3	I don't think that public postings on social media affects my professional image.
Availability	AVA1	I think social media can facilitate my work.
	AVA2	I think social media makes it easy for me to contact my clients.
	AVA3	I think using social media makes it easier for my clients to contact me.
Symbol variety	SYM1	I think it is more friendly to have emoticons in conversations with my clients.
	SYM2	Only use text to online talking is not enough to express my emotions or feelings when I use social media.
	SYM3	I use other features in social media (e.g., emoticons, images, video clips) to express emotions when communicating with clients.
	SYM4	When using social media to communicate with clients, I am aware of the options for using other features of social media (e.g., emojis, images, videos).
Social-oriented usage of social media	SOC1	I can make friends in the organization through social media.
	SOC2	I can find like-minded people through social media.
	SOC3	I can meet new friends through social media.
	SOC4	I use social media in order to meet friends/clients I have never met.
Work-oriented usage of social media	WOR1	I discuss work with colleagues through social media.
	WOR2	I use social media to contact customers.
	WOR3	I use social media for work.
	WOR4	For work, I think social media is one of the tools that must be used.
Work efficiency	WEF1	After using social media, my daily work is more efficient.
	WEF2	I am able to communicate with my clients better through social media.
	WEF3	I think it is more efficient to use social media to communicate with clients.
